# Non-Steroidal Anti-inflammatory Drugs Decrease E2F1 Expression and Inhibit Cell Growth in Ovarian Cancer Cells

**DOI:** 10.1371/journal.pone.0061836

**Published:** 2013-04-24

**Authors:** Blanca L. Valle, Theresa D'Souza, Kevin G. Becker, William H. Wood, Yongqing Zhang, Robert P. Wersto, Patrice J. Morin

**Affiliations:** 1 Laboratory of Molecular Biology and Immunology, National Institute on Aging, Baltimore, Maryland, United States of America; 2 Research Resources Branch, National Institute on Aging, NIH, Baltimore, Maryland, United States of America; 3 Department of Pathology, Johns Hopkins Medical Institutions, Baltimore, Maryland, United States of America; Mayo Clinic, United States of America

## Abstract

Epidemiological studies have shown that the regular use of non-steroidal anti-inflammatory (NSAIDs) drugs is associated with a reduced risk of various cancers. In addition, in vitro and experiments in mouse models have demonstrated that NSAIDs decrease tumor initiation and/or progression of several cancers. However, there are limited preclinical studies investigating the effects of NSAIDs in ovarian cancer. Here, we have studied the effects of two NSAIDs, diclofenac and indomethacin, in ovarian cancer cell lines and in a xenograft mouse model. Diclofenac and indomethacin treatment decreased cell growth by inducing cell cycle arrest and apoptosis. In addition, diclofenac and indomethacin reduced tumor volume in a xenograft model of ovarian cancer. To identify possible molecular pathways mediating the effects of NSAID treatment in ovarian cancer, we performed microarray analysis of ovarian cancer cells treated with indomethacin or diclofenac. Interestingly, several of the genes found downregulated following diclofenac or indomethacin treatment are transcriptional target genes of E2F1. E2F1 was downregulated at the mRNA and protein level upon treatment with diclofenac and indomethacin, and overexpression of E2F1 rescued cells from the growth inhibitory effects of diclofenac and indomethacin. In conclusion, NSAIDs diclofenac and indomethacin exert an anti-proliferative effect in ovarian cancer in vitro and in vivo and the effects of NSAIDs may be mediated, in part, by downregulation of E2F1.

## Introduction

Ovarian cancer is the leading cause of death by gynecological malignancies. When detected early, the 5-year survival rate is as high as 90%, but unfortunately, the vast majority of cases are diagnosed as late-stage disease, which is often resistant to conventional chemotherapy. Consequently, the overall 5-year survival rate of ovarian cancer is approximately 30–40%. It is therefore imperative to investigate new approaches for the treatment and management of this deadly disease.

Epidemiological studies have suggested that the regular use of non-steroidal anti-inflammatory (NSAIDs) drugs is associated with a reduced risk of various cancers, including colorectal, breast, lung and ovarian cancers [1], [2], [3]. In addition, in vitro and animal studies have shown that NSAIDs can decrease the initiation and/or progression of several cancers [4], [5], [6]. For example, the NSAID indomethacin inhibited the growth of chemically-induced colon cancers in rats [7], [8]. In addition, indomethacin reduced the growth of new and established spontaneous mammary tumors [9]. The NSAID diclofenac decreased the growth of pancreatic and non-small cell lung cancer xenografts [10], [11]. However, there are limited preclinical studies investigating the effects and mechanisms of action of diclofenac and indomethacin in ovarian cancer [12], [13]. In this regard, Zerbini et. al. reported that diclofenac decreased tumor volume in SCID mice with ovarian cancer cell SKOV-3 xenografts by ∼20% [12]. However, another study reported that indomethacin had no effect on the growth of ovarian reticular cell sarcoma M5076 [13].

To our knowledge, there are no reports on the effects of indomethacin specifically in epithelial ovarian cancer, which comprises the majority of ovarian cancers (approximately 90%). In this study, we have investigated the effects of the NSAIDs diclofenac and indomethacin in ovarian cancer cells. We report that NSAIDs significantly reduced ovarian cancer cell growth in vitro and in vivo, and, using microarray analysis, we identified the transcription factor E2F1 as a mediator of this effect. Importantly we found that ectopic E2F1 expression reversed the growth-inhibitory effects of NSAIDs suggesting that NSAIDs could act in part through a mechanism involving E2F1 downregulation in ovarian cancer cells.

## Materials and Methods

### Ethics statement

All procedures performed in mice were approved by the Institutional Animal Care and Use Committee of the National Institute on Aging. This study was performed in accordance with the Guide for the Care and Use of Laboratory Animals of the National Institutes of Health.

### Reagents

Diclofenac and indomethacin were purchased from Sigma-Aldrich (St. Louis, MO). Anti-E2F1, anti-E2F4, anti-MCM2, and anti-MCM4 antibodies were purchased from Proteintech (Chicago, IL). Antibodies from Abcam (Cambridge, MA) recognized GAPDH, and antibodies from Cell Signaling (Danvers, MA) recognized Rb. E2F1 and eGFP expression plasmids were from GeneCopoeia (Rockville, MD). Rb siRNA (sc-29468) was from Santa Cruz Biotechnology (Santa Cruz, CA).

### Cell culture and transfections

The serous ovarian adenocarcinoma cell lines HEY, OVCAR5 and UCI-101 were kindly provided as follows: Hey cells by Dr. Robert C. Bast [14], OVCAR5 cells by Dr. Thomas C. Hamilton [15] and UCI-101 cells by Dr. Michael J. Birrer [16]. Cells were cultured in McCoy's 5A culture media supplemented with 10% fetal bovine serum and Pen/Strep (100 units/mL penicillin and 100 µg/mL streptomycin), and incubated at 37°C in an atmosphere of 5% CO_2_. All experiments were performed in serum-containing media.

For transfections, HEY cells were seeded at a density of 5×10^4^ cells in 12-well plates and transiently transfected with E2F1 or eGFP as control; or Rb siRNA or control siRNA for 24 hours, using X-treme Gene 9 DNA Transfection Reagent (Roche) or Lipofectamine 2000 (Invitrogen), according to manufacturer's instructions. Cells were then replated at densities of 5×10^3^ cells in 6-well plates and treated for 24–48 hours with 300 µM diclofenac or indomethacin. Cells were then lysed and analyzed by immunoblotting or washed and used in clonogenic assays.

### MTS and clonogenic assays

For MTS viability assays (Promega), HEY, OVCAR5 or UCI-101 cells were seeded at a density of 7×10^3^ cells in 96-well plates and treated for 48 hours with 300 µM diclofenac or indomethacin. Cells were then incubated with MTS reagents and absorbance at 490 nm was measured after 1–4 hr incubation at 37°C. For dose-dependence viability assays, cells were treated for 24 hours with increasing concentrations of diclofenac or indomethacin (50, 100, 250, and 500 µM). For clonogenic assays, HEY, OVCAR5 or UCI-101 cells were seeded at a density of 3×10^3^ cells in 6-well plates and treated for 48 hours with 300 µM diclofenac or indomethacin, washed and allowed to grow for 10 days. Colonies were then visualized by staining with crystal violet (0.5% crystal violet in 50% methanol).

### Cell cycle and apoptosis analysis

and treated for 24 or 48 hours with 300 µM diclofenac or indomethacin. Cells were labeled with propidium iodide and analyzed in a FACS Caliber Flow Cytometer (Becton Dickinson, Franklin Lakes, NJ). For analyzing apoptosis, cells were treated for 48 or 96 hours with 300 µM diclofenac or indomethacin. Apoptosis and DNA content were assayed by BrdU and propidium iodide staining using an Apo-BrdU kit (Phoenix Flow Systems, San Diego, CA) and analyzed in a LSR-II Flow Cytometer (BD Biosciences, San Jose, CA).

### Animal studies

Female 7–8 week athymic nude mice were obtained from Harlan Laboratories (Frederick, MD) and all experiments were reviewed and approved by the NIA IACUC.

HEY cells (1.5×10^6^) were injected subcutaneously into both flanks of nude mice. For the diclofenac study, mice were randomized into 2 groups: control (PBS) or diclofenac (18 mg/kg); 6 mice per group. Mice were injected intraperitoneally twice a week for 4 weeks starting 3 days after cell injection. Tumors were measured twice a week, starting at week 1, using calipers and W^2^L/2 [17] was used to calculate the tumor volume. For the indomethacin study, mice were randomized into 2 groups: control (water) or indomethacin (2.5 mg/kg); 5 mice per group. Indomethacin was given daily in drinking water for 6 weeks, starting the day after cell injection. Tumors were measured twice a week, starting at week 3, using calipers and W^2^L/2 [17] was used to calculate the tumor volume. Data is represented as means ± standard errors. The concentrations of diclofenac and indomethacin used for this study are similar to others that have been previously shown to be effective and well-tolerated in mice [4], [10], [11], [18].

### Microarray analysis

HEY, OVCAR5 and UCI-101 cells were seeded in 60 mm dishes and treated for 24 hours with 300 µM diclofenac or indomethacin. RNA was isolated using the RNeasy kit (Qiagen, Valencia, CA). Total RNA was used to generate biotin labeled cDNA using the Illumina TotalPrep RNA Amplification Kit (Ambion, Grand Island, NY) and analyzed by Illumina microarrays (Illumina's Sentrix HumanRef-8 Expression BeadChips) in the Microarray Core Facility (National Institute on Aging). Each BeadChip has 24,000 well-annotated RefSeq transcripts with an average of 30-fold redundancy. Genes that were differentially expressed 2-fold or more in all three cell lines were identified and a subset of these genes selected for further analysis. The microarray data has been submitted to the NCBI Gene Expression Omnibus (GEO) database (Accession number GSE45052).

### Real-Time RT-PCR

HEY, OVCAR5, and UCI-101 cells were treated for 24 hr with 300 µM diclofenac or indomethacin. RNA was isolated using the RNeasy kit and 1 µg of total RNA was used to generate cDNA using Taqman Reverse Transcription Reagents (PE Applied Biosystems). For PCR amplification and quantitation, the SYBR Green I assay and the GeneAmp 7300 Sequence Detection System (PE Applied Biosystems) were used. The comparative CT method was used to determine the relative expression level of genes in each NSAID treated sample to the CT value of untreated sample (PE Applied Biosystems) and GAPDH values were used for normalization [19]. The sequences of the primers are available from the authors.

### Immunoblotting

Cells were washed and cell lysates were prepared in lysis buffer (150 mM Tris-HCl, pH 6.8, 25% glycerol, and 5% SDS). Cell lysates were separated by SDS-PAGE on 4–12 or 10–20% Tris-Glycine gels and transferred to PVDF membranes. The membranes were blocked with TBS-T + 5% non-fat dry milk and incubated overnight at 4°C with antibodies specific for the indicated proteins. After washing the membranes, they were incubated with horseradish-peroxidase conjugated secondary antibodies. Protein detection was performed by enhanced chemiluminescence (Amersham, Pittsburgh, PA).

### Statistics

Wilcoxon signed rank test was performed to determine significance in the mouse studies. For all other analyses, One-way ANOVA and Dunnett's multiple comparison test was performed. All statistical analyses were done using Graph Pad Prism 3 software.

## Results

### NSAIDs decrease viability of ovarian cancer cells in a dose-dependent manner

**Figure 1:**
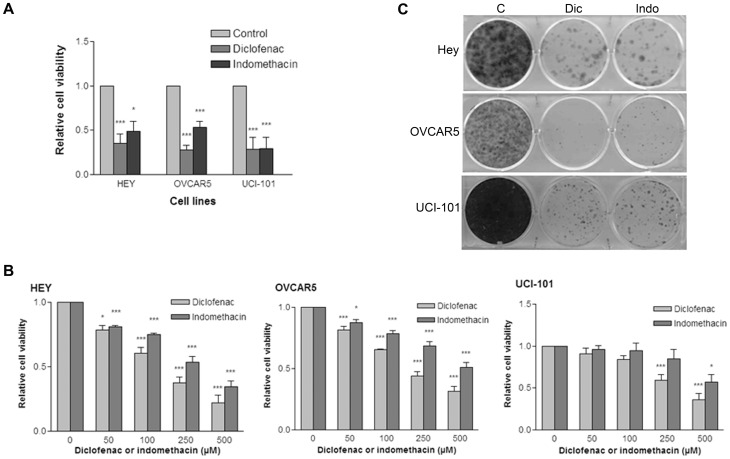
NSAIDs decrease the viability of ovarian cancer cells in a dose-dependent manner. A) HEY, OVCAR5 or UCI-101 cells were treated for 48 hours with 300 µM diclofenac or indomethacin. Cells were then incubated with MTS reagents and absorbance at 490 nm was measured after 1 hr incubation at 37°C. The average of 3 experiments is shown. Statistical significance is represented as follows: * p<0.05 and *** p<0.001. B) Cells were treated for 24 hours with the indicated concentrations of diclofenac or indomethacin. Cell viability was measured using an MTS assay. The average of 3 experiments is shown. Statistical significance is represented as follows: * p<0.05 and *** p<0.001. C) Cells were treated for 48 hours with 300 µM diclofenac or indomethacin, washed and allowed to grow for a total of 7 to 14 days. Cells were then stained with crystal violet. Image shown is representative of 3 experiments.

To determine whether NSAIDs could reduce growth of ovarian cancer cells in vitro, we treated the cell lines HEY, OVCAR5, and UCI-101 with the NSAIDs diclofenac and indomethacin. After 48 hours of treatment, diclofenac and indomethacin reduced cell numbers by more than 50% in the treated cell lines relative to untreated populations in three different ovarian cancer cell lines (Figure 1A). The effects of these NSAIDs were dose-dependent, as increasing concentrations of diclofenac or indomethacin further decreased the number of viable cells (Figure 1B). In addition, diclofenac and indomethacin decreased the colony-forming ability of ovarian cancer cells (Figure 1C).

### Diclofenac and Indomethacin promote cell cycle arrest and apoptosis in ovarian cancer cells

**Figure 2:**
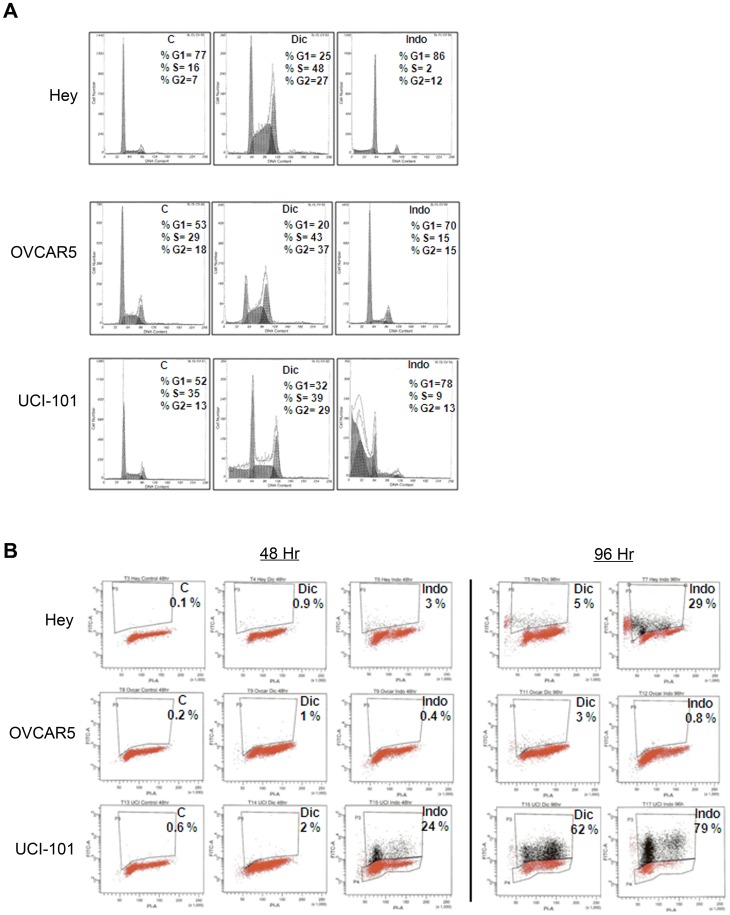
Diclofenac and Indomethacin promote cell cycle arrest and apoptosis in ovarian cancer cells. A) HEY, OVCAR5 and UCI-101 cells were treated for 24 hours with 300 µM diclofenac or indomethacin. Cells were labeled with propidium iodide and analyzed in a FACS Caliber Flow Cytometer. Image shown is representative of 4 experiments. B) Cells were treated for 48 or 96 hours with 300 µM diclofenac or indomethacin. Apoptosis and DNA content were assayed by BrdU and propidium iodide staining using Apo-BrdU kit. Cells in upper quadrant box (P3 gate) represent the apoptotic population.

Since diclofenac and indomethacin reduced ovarian cancer cell growth, we wanted to determine whether this decrease was due to alterations in the cell cycle. Diclofenac and indomethacin induced cell cycle arrest in all 3 ovarian cancer cell lines examined, as detected by propidium iodide staining after 48 hours of treatment (Figure 2A). The two NSAIDs had different effects on the cell cycle, as diclofenac induced an accumulation of cells at S phase and G2, while indomethacin induced G1 arrest in all three ovarian cancer cell lines examined. We also observed a sub-G1 population of cells in the UCI-101 cells treated with diclofenac and indomethacin and in HEY cells treated with indomethacin, suggesting increased apoptosis. To determine whether these cells undergo apoptosis upon treatment with NSAIDs, we treated cells with diclofenac or indomethacin for 48 or 96 hours and measured DNA fragmentation by Apo-BrdU staining. As shown in Figure 2B, UCI-101 cells undergo apoptosis upon treatment with both NSAIDs, while HEY cells undergo apoptosis only with indomethacin (Figure 2B). OVCAR5 does not undergo apoptosis after 4 days of treatment with NSAIDs. Taken together, these results suggest that NSAIDs reduce ovarian cancer cell growth by inducing cell cycle arrest and apoptosis.

### NSAIDs reduce growth of HEY xenografts in nude mice

**Figure 3:**
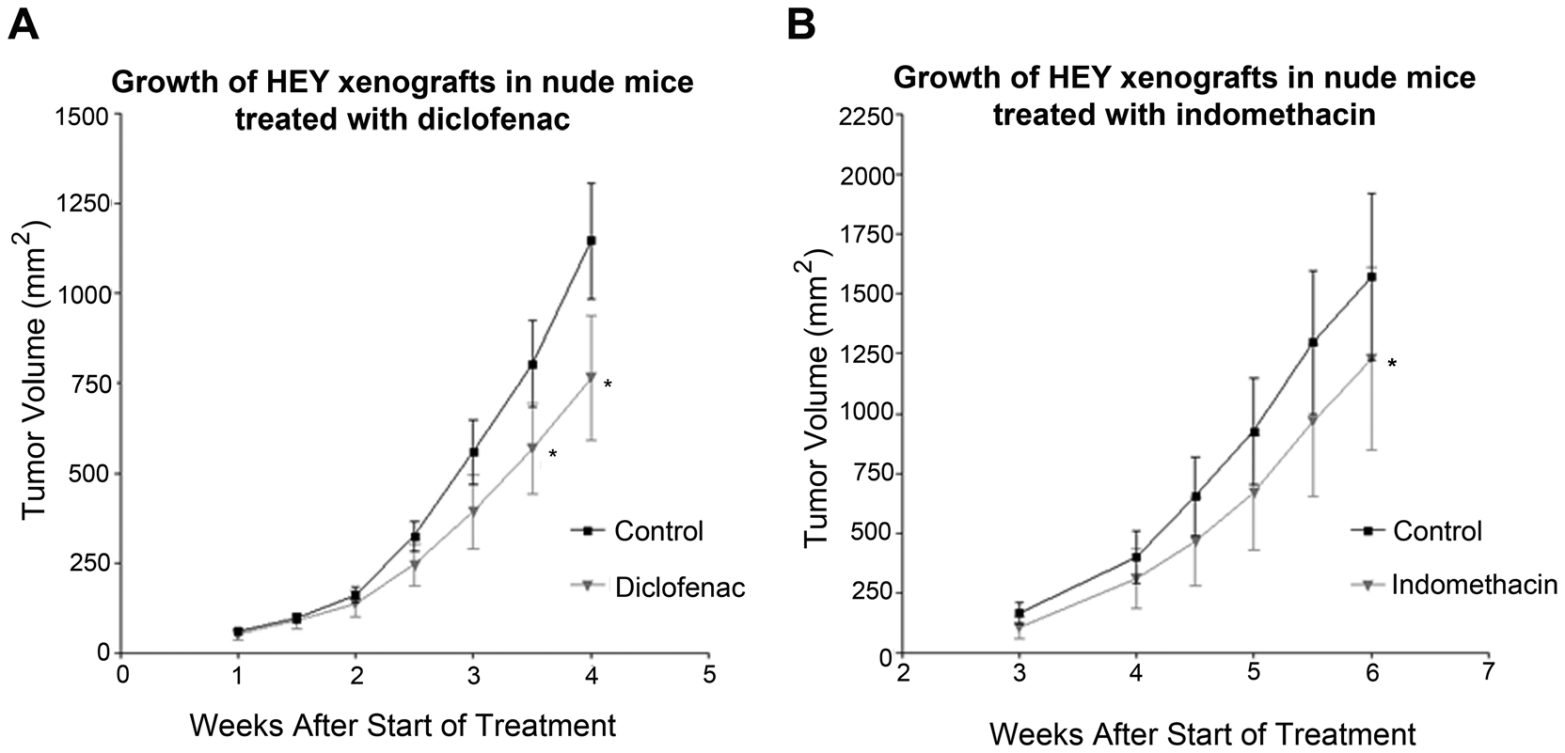
NSAIDs reduce the growth of HEY xenografts in nude mice. HEY cells (1.5×10^6^) were injected subcutaneously into both flanks of nude mice. A) Mice were randomized into 2 groups: control or diclofenac (18 mg/kg). Treatment was started three days after cell injection and was administered intraperitoneally twice a week for 4 weeks. Tumors were measured twice a week, starting at week 1. The control group (only PBS) consisted of 6 mice that developed 12 tumors in total; the diclofenac group (18 mg/kg) consisted of 6 mice that developed a total of 11 tumors. Values represent means ± standard errors. Statistical significance is represented as * p<0.05. B) Mice were randomized into 2 groups: control or indomethacin (2.5 mg/kg). Treatment was started the day after cell injection and was given daily in drinking water for 6 weeks. Tumors were measured twice a week, starting at week 3. The control group (only water) consisted of 5 mice that developed 9 tumors in total; the indomethacin group (2.5 mg/kg) consisted of 5 mice that developed a total of 5 tumors. Values represent means ± standard errors. Statistical significance is represented as * p<0.05.

To determine whether NSAIDs could inhibit tumor growth in vivo, we used an ovarian cancer xenograft mouse model in nude mice. HEY cells were injected subcutaneously into both flanks of nude mice and randomized into 2 groups: control (PBS) or diclofenac (18 mg/kg), injected intraperitoneally twice a week, for 4 weeks. As observed, diclofenac treatment reduced significantly (p = 0.016) the growth of HEY xenografts in nude mice (Figure 3A). The average tumor volume in the diclofenac-treated group was reduced by 33%, as compared to the control group. Alternatively, mice were randomized into control (water) or indomethacin (2.5 mg/kg) groups, and drug administered daily in drinking water for 6 weeks. As observed, indomethacin significantly (p = 0.031) reduced the growth of HEY xenografts in nude mice (Figure 3B). The average tumor volume was reduced significantly by 22% in the group treated with indomethacin as compared to the control group. In addition, mice in the indomethacin treated group developed fewer tumors than the control group, 5 and 9 respectively. Taken together, these results indicate that NSAIDs decrease ovarian cancer cell growth in vivo.

### E2F1 and target genes are downregulated in cells treated with NSAIDs

**Figure 4:**
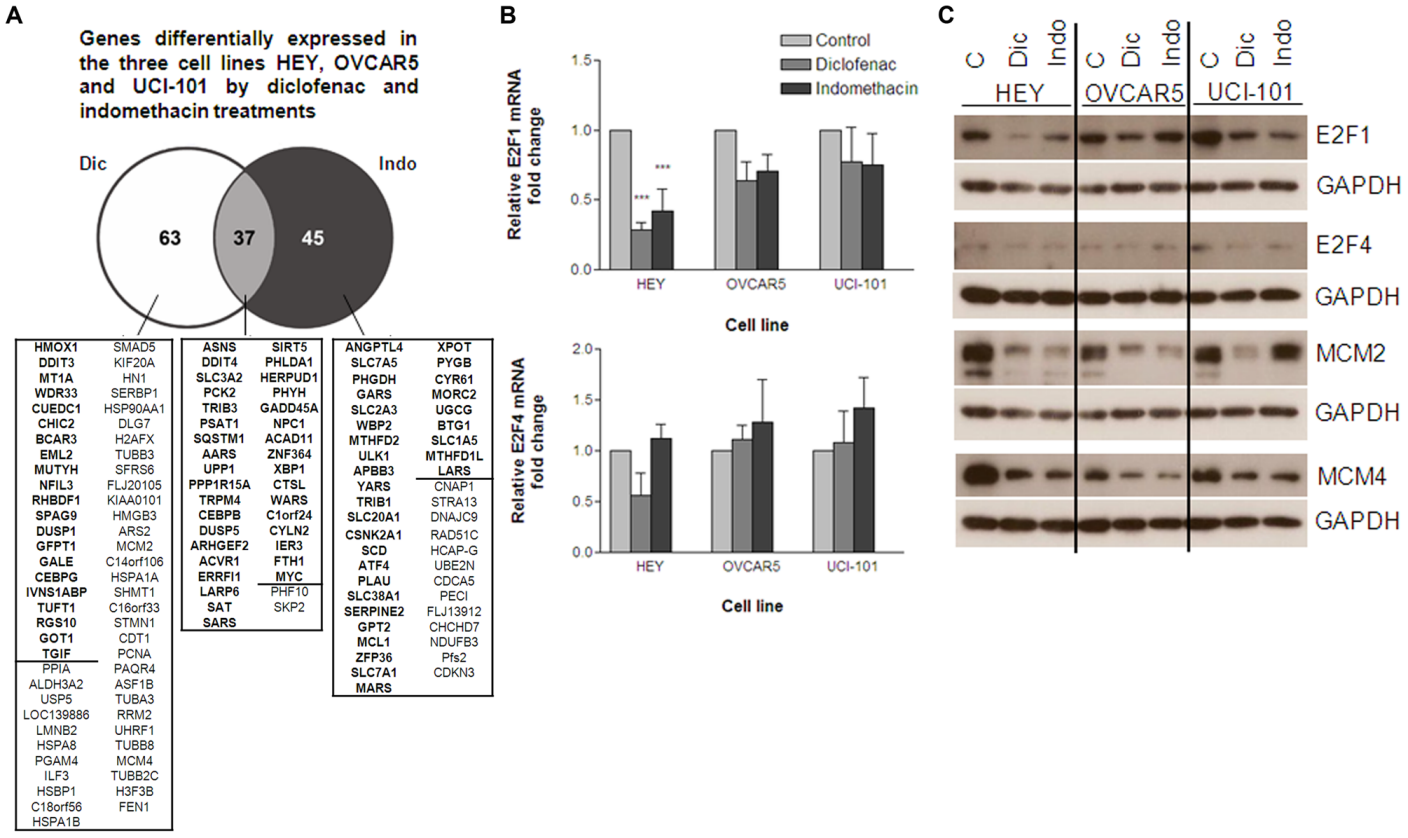
E2F1 and target genes are downregulated in cells treated with NSAIDs. HEY, OVCAR5 and UCI-101 cells were treated for 24 hours with 300 µM diclofenac or indomethacin. A) RNA was analyzed by Illumina microarrays. The number of mRNAs that were differentially expressed 2-fold or more in all three cell lines is indicated for the diclofenac and indomethacin treatments (Shown in bold are the overexpressed mRNAs; shown in regular font are the downregulated mRNAs). B) RNA from the indicated cell lines treated with diclofenac or indomethacin was analyzed by Real-time RT-PCR. E2F1 and E2F4 mRNA levels are expressed as fold change in treated samples as compared to the untreated, after normalization with GAPDH. The average of 3 experiments is shown. Statistical significance is represented as *** p<0.001. C) Cell lysates of the indicated cell lines that were treated with diclofenac or indomethacin were analyzed by immunoblotting with antibodies specific for the indicated proteins. GAPDH was used as a protein loading control.

To investigate the genes differentially expressed in ovarian cancer cells after treatment with NSAIDs, we treated HEY, UCI-101, and OVCAR5 cells with diclofenac and indomethacin for 24 hours and performed microarray analysis. The mRNAs differentially expressed (≥2-fold) in the three cell lines are shown (Figure 4A). A small subset of differentially expressed mRNAs was shared between the three cell lines, and a small number of differentially expressed mRNAs were shared by treatment with diclofenac and indomethacin in all three cell lines.

The majority of mRNAs differentially expressed upon treatment with diclofenac were found to be downregulated, while the majority of genes affected by indomethacin were upregulated. Given that diclofenac and indomethacin induced cell cycle arrest and apoptosis, we chose for validation genes possibly involved in these processes. We confirmed by quantitative RT-PCR, that the expression of *MCM2*, *MCM4*, and other mRNAs encoding proteins involved in DNA replication and cell cycle regulation were downregulated by diclofenac (Table 1), while *GADD45A* and *PPP1R15A* mRNAs, among others encoding apoptosis-relevant proteins, were upregulated by indomethacin and by diclofenac (Table 1). In addition, we validated mRNAs upregulated by indomethacin encoding solute carrier proteins SLC1A5 and SLC7A1 (Table 1). However, mRNAs that showed differential expression levels only by diclofenac or indomethacin in the microarray analysis, were found differentially expressed by both drugs when assayed by RT-qPCR (Table 1).

**Table 1:** Expression levels of target genes by Real-Time RT-PCR.

	Gene	Hey		OVCAR5		UCI-101	
Expression in microarray		Dic	Indo	Dic	Indo	Dic	Indo
↓ Diclofenac	**MCM2**	0.3^†^	0.5^†^	0.4^†^	0.5^†^	0.5*	0.5*
	**MCM4**	0.1^†^	0.2^†^	0.2^†^	0.3^†^	0.4^†^	0.4^†^
	**CDT1**	0.5*	0.7	0.4^†^	0.6*	0.8	0.8
	**PCNA1**	0.3^†^	0.4^†^	0.2^†^	0.4^†^	0.4	0.6
	**RRM2**	0.3^†^	0.3^†^	0.2^†^	0.3^†^	0.6	0.5*
	**KIAA0101**	0.8	0.5^†^	0.5*	0.7	0.7	0.3*
↑ Indomethacin	**SLC1A5**	1.7	2.3*	2.6^†^	2.8^†^	2.1	2.1
	**SLC7A1**	0.9	0.7	1.0	1.0	1.1	0.7
↑ Diclofenac/Indomethacin	**PPP1R15A**	4.4	6.7*	8.3^†^	5.6^†^	13.2	17.6
	**GADD45A**	3.1*	2.5	4.1^†^	2.6^†^	11.7	8.0
	**XBP1**	1.2	2.4	1.8	2.3	1.2	1.7

HEY, OVCAR5 and UCI-101 cells were treated for 24 hours with 300 µM of diclofenac or indomethacin. RNA was isolated and RT-qPCR performed. Shown is the average of 3 independent experiments. Numbers reflect the fold change difference in the mRNA levels in the treated samples, as compared to the untreated samples, after normalization with GAPDH. Statistical significance is represented as * p<0.05; ^†^ p<0.001.

To identify transcription factors that might regulate the differential expression of these mRNAs, transcription factor target analysis was performed using WebGestalt. WebGestalt (WEB-based GEne SeT AnaLysis Toolkit) is an online tool (http://bioinfo.vanderbilt.edu/webgestalt/) to analyze gene sets and the transcription factor target analysis program of WebGestalt predicts transcription factors likely involved in the regulation of these genes. Table S1 shows the most highly significant transcription factors predicted, along with the corresponding differentially expressed genes identified by microarray. E2F1, nuclear factor Y and E2F4 were the top transcription factors predicted to regulate the genes differentially expressed by treatment with diclofenac, and the corresponding mRNAs were downregulated. NFAT and AP1 were the top two transcription factors predicted to regulate the genes affected by indomethacin, and the mRNAs encoding these proteins were mostly upregulated.

To determine whether NSAIDs had an effect on the expression of the transcription factors identified above, we examined the mRNA and protein levels of transcription factors E2F1, NFY, E2F4, NFAT and AP1. In agreement with the trend reported above, *E2F1* mRNA was downregulated in the three ovarian cancer cell lines after treatment for 24 hours with diclofenac or indomethacin, and particularly in HEY cells (Figure 4B). E2F1 protein was also downregulated, as well as MCM2 and MCM4 proteins (Figure 4C). *NFAT* and *AP1* mRNAs were mostly upregulated (Table S2), as were their transcriptional targets. However, NFAT and AP1 protein abundance was lower (Figure S1), despite showing higher mRNA levels. The levels of *NFY* and *E2F4* mRNAs were not significantly changed (Figure 4B and Table S2).

### E2F1 overexpression increases the survival of NSAID-treated ovarian cancer cells

**Figure 5:**
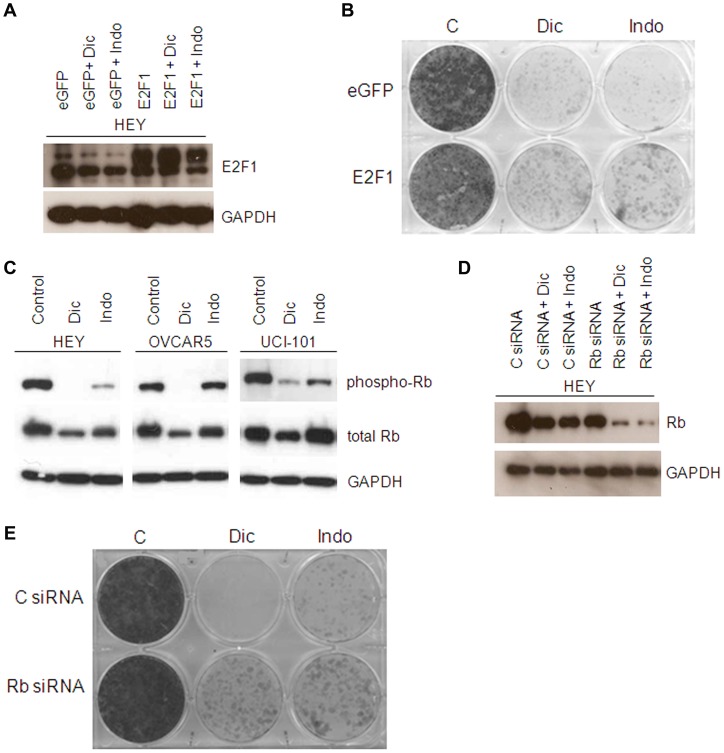
E2F1 overexpression increases the survival of NSAID treated ovarian cancer cells. A) HEY cells were transiently transfected with E2F1 or eGFP as a control and treated for 24 hours with 300 µM diclofenac or indomethacin. Cell lysates were analyzed by immunoblotting with the indicated antibodies. B) HEY cells were transiently transfected with E2F1 or eGFP as a control and treated for 48 hours with 300 µM diclofenac or indomethacin. Cells were then washed and allowed to grow and stained with crystal violet. Image shown is representative of 2 experiments. C) HEY cells were treated for 24 hours with 300 µM diclofenac or indomethacin. Cell lysates were analyzed by immunoblotting. D) HEY cells were transfected with Rb siRNA and treated for 24 hours with diclofenac or indomethacin. Cell lysates were analyzed by immunoblotting. E) HEY cells were transfected with Rb siRNA and treated for 48 hours with 300 µM diclofenac or indomethacin, cells were then washed and allowed to grow and stained with crystal violet. Image shown is representative of 3 experiments.

Since the expression of both E2F1 and its target genes is downregulated upon treatment with diclofenac and indomethacin, we wanted to determine whether E2F1 was involved in the growth inhibitory effects of NSAIDs in ovarian cancer cells. We overexpressed E2F1 in HEY cells (Figure 5A) and observed an increased survival following treatment with diclofenac and indomethacin (Figure 5B). This experiment suggests that downregulation of E2F1 might be involved in inhibition of cell growth by NSAIDs.

Retinoblastoma protein (Rb) blocks cell cycle progression by inhibiting E2F1 and blocking subsequent transcriptional activation of genes important for entry into S phase. We found that levels of phosphorylated Rb were decreased upon treatment with NSAIDs (Figure 5C), indicative of Rb activation. Therefore, we sought to downregulate the expression of Rb to further investigate whether E2F1 was involved in the effects of NSAIDs (Figure 5D), as silencing Rb using small interfering (si) RNA should mimic the activation of E2F1. Treatment with Rb siRNA increased the survival of HEY cells treated with diclofenac and indomethacin (Figure 5E), suggesting that E2F1 inhibition or downregulation could be involved in the growth-inhibitory effects of NSAIDs in these cells.

## Discussion

In this study, we have evaluated the effects of the NSAIDs diclofenac and indomethacin in ovarian cancer cells and a mouse xenograft model. To our knowledge, there are no reports about the effects of indomethacin in vivo in epithelial ovarian cancer. Here we report that indomethacin decreased significantly the growth of HEY xenografts by 22% in nude mice, suggesting that indomethacin might reduce progression of ovarian cancer in vivo. In addition, we report that diclofenac reduced significantly the growth of HEY xenografts. This is consistent with a recent study reporting that diclofenac reduced the growth of ovarian cancer cell SKOV-3 xenografts [12]. Although our results are in agreement, in our study we found a slightly better effect of diclofenac (33% compared to 20%) and the effects were noticeable earlier on (after 4 weeks treatment vs 8 weeks). This could be due in part to differences in the mode of delivery (intraperitoneal vs in diet). Taken together, our data demonstrates that these NSAIDs have growth inhibitory effects in vivo.

Indomethacin and diclofenac have been shown to induce cell cycle arrest and apoptosis in several cancer cell types [12], [20], [21], [22], [23], [24]. In our study, indomethacin and diclofenac reduced ovarian cancer cell growth, at least in part through the induction of cell cycle arrest in the three ovarian cancer cell lines examined. Diclofenac and indomethacin had different effects on the cell cycle, while indomethacin induced a G1 arrest, diclofenac induced an accumulation of cells at S phase and G2 arrest. The results of indomethacin are consistent with previous studies in which indomethacin has also been reported to induce G1 arrest in a variety of cells [20], [21], [22], [25]. However, only two previous studies have reported effects of diclofenac on the cell cycle: in smooth muscle cells, diclofenac induced G1 arrest [22], while in murine glioma cells diclofenac induced G2 arrest [26]. In our study, diclofenac induced an accumulation of cells at S phase and G2 arrest.

To address the difference we observed in cell cycle effects between diclofenac and indomethacin, we attempted to identify differences in gene expression following treatment with each drug. Among the mRNAs validated by RT-qPCR, we found that the majority of the genes altered by diclofenac treatment were also differentially expressed in the indomethacin-treated cells. Therefore, further studies of these genes are warranted to determine what causes the differences in cell cycle effects by diclofenac and indomethacin.

WebGestalt Transcription Factor analysis of the genes differentially expressed by diclofenac and indomethacin predicted that several of the genes downregulated by diclofenac are target genes of E2F1. E2F1 is a transcription factor that controls cell cycle, by regulating the expression of genes necessary for entry into S phase. E2F1 is overexpressed in a number of cancers, and its overexpression has been generally associated with a poor prognosis [27], [28], [29], [30]. In women with ovarian cancer, overexpression of E2F1 has been associated with decreased disease-free survival and decreased overall survival [31], [32]. In previous studies, the downregulation of expression and/or activity of E2F1 by NSAIDs was thought to contribute to the growth-inhibitory effects of NSAIDs [20], [33], [34], [35], [36], [37]. It has been reported that the activity of E2F1 is downregulated by NSAIDs celecoxib and sulindac [20]. In the same study, indomethacin did not decrease the activity of E2F1 in the head and neck squamous carcinoma cell line UM-SCC-1. Here, we report that E2F1 was downregulated at the mRNA and protein levels in the three ovarian cancer cell lines examined by treatment with diclofenac and indomethacin. Overexpression of E2F1 in HEY cells rescued cells from the anti-proliferative effects of NSAIDs leading to increased survival, suggesting that E2F1 mediates in part the effects of NSAIDs. These results suggest that the use of NSAIDs could be therapeutically relevant, in particular for a subset of ovarian cancers overexpressing E2F1, and which are predicted to have a poor prognosis.

Among the transcripts differentially expressed in diclofenac- and indomethacin-treated cells were *MCM2*, *MCM4*, *PCNA* and *RRM2* mRNAs, all of them targets of E2F1 and important for replication and cell cycle progression. Recently, it was reported that RRM2, PCNA and MCM2 were downregulated upon treatment with the NSAID NS-398 in pancreatic cancer cells [38]. Downregulation of MCM2, and other MCM proteins had been reported to induce G1 and G2 arrests [39], [40] contributing to cellular growth inhibition. Therefore, it is possible that downregulation of these proteins could contribute to the anti-proliferative effects of NSAIDs in ovarian cancer cells in our study.

In conclusion, we show that diclofenac and indomethacin inhibit ovarian cancer cell growth in vitro and in an animal model, by inducing cell cycle arrest and apoptosis. The growth inhibitory effects of diclofenac and indomethacin are mediated in part by a mechanism that involves downregulation of E2F1. Taken together, our results support the view that the use of NSAIDs could be therapeutically beneficial for ovarian cancer.

## Supporting Information

Figure S1 **Protein levels of transcription factors NFAT and AP1.** HEY, OVCAR5 and UCI-101 cells were treated for 24 hours with diclofenac (300 µM) or indomethacin (300 µM). Cell lysates were analyzed by immunoblotting with antibodies specific for the indicated proteins.
(TIF)

Table S1 **Transcription factors predicted by WebGestalt to regulate differentially expressed genes in diclofenac and indomethacin treated cells.** Transcription Factor Target Analysis of the differentially expressed gene sets was performed using WebGestalt (http://bioinfo.vanderbilt.edu/webgestalt/) to identify the transcription factors possibly involved in the regulation of the differentially expressed genes. Shown are the top transcription factors (highest significance) predicted along with the corresponding genes. Genes overexpressed are shown in bold and genes downregulated are shown in regular font.
(TIF)

Table S2 **Relative mRNA levels of transcription factors by Real-Time RT-PCR.** Numbers reflect the fold change difference in the mRNA levels in the treated samples, as compared to the untreated samples, after normalization with GAPDH. Shown is the average of 3 independent experiments. One-way ANOVA and Dunnett's multiple comparison test was performed using Graph Pad Prism 3 software and statistical significance is represented as * p<0.05.
(TIF)
