# Supplementary figures and images for: Non-Steroidal Anti-inflammatory Drugs Decrease E2F1 Expression and Inhibit Cell Growth in Ovarian Cancer Cells
Source: PLoS One. 2013 Apr 24;8(4):e61836. doi: 10.1371/journal.pone.0061836 (PMC3634839; doi:10.1371/journal.pone.0061836)

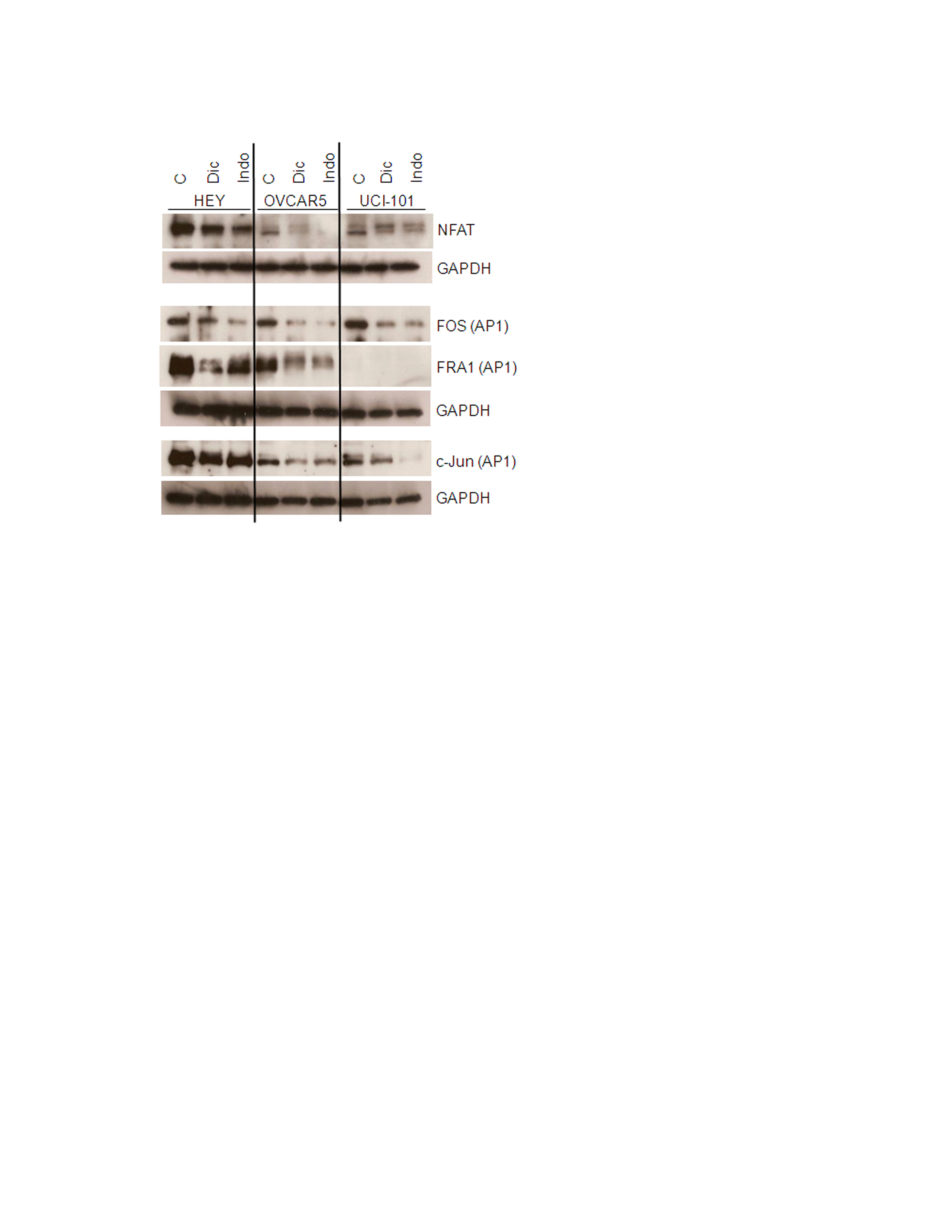

Supplement: Figure S1 — Protein levels of transcription factors NFAT and AP1. HEY, OVCAR5 and UCI-101 cells were treated for 24 hours with diclofenac (300 µM) or indomethacin (300 µM). Cell lysates were analyzed by immunoblotting with antibodies specific for the indicated proteins. (TIF) [file pone.0061836.s001.tif]

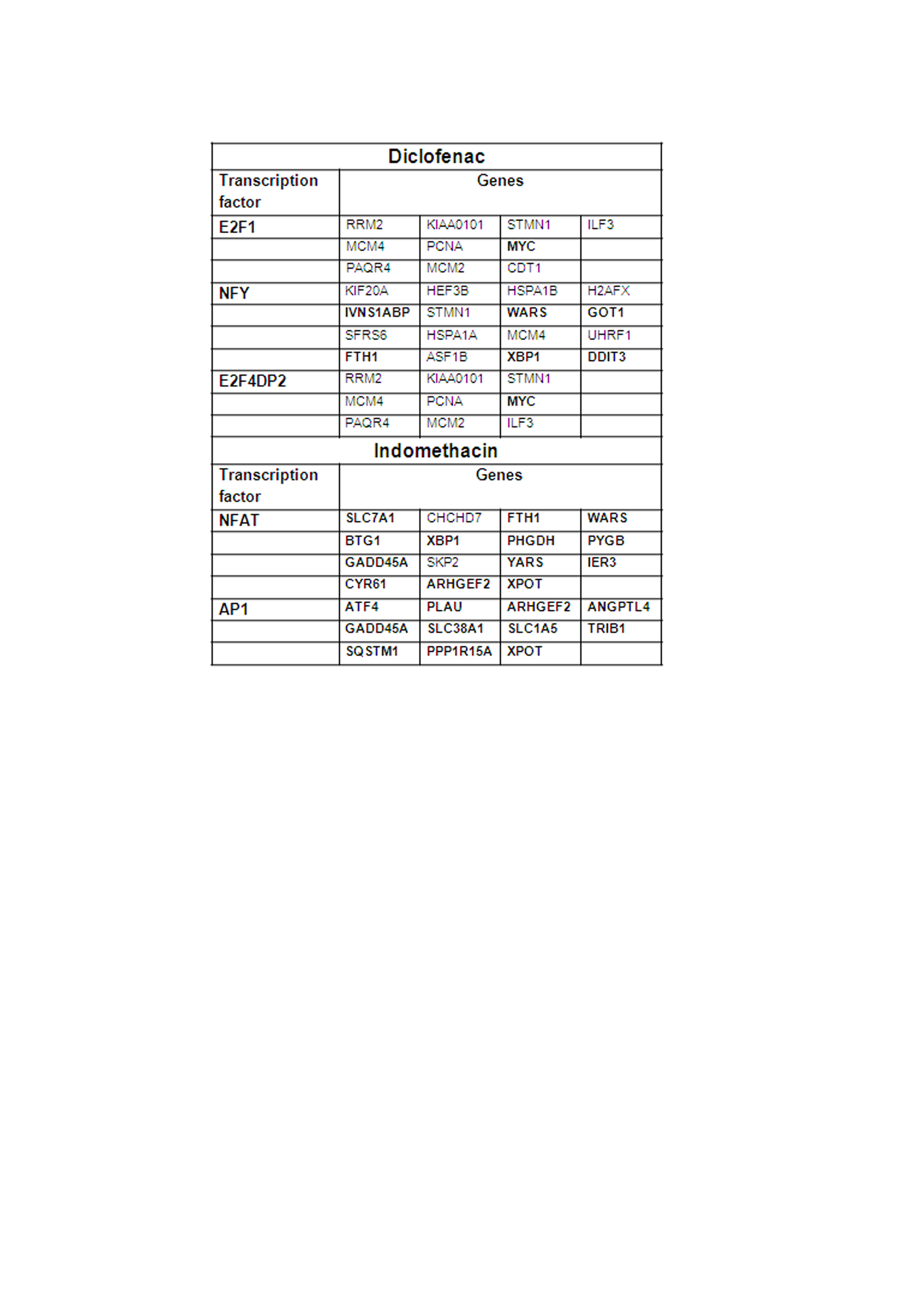

Supplement: Table S1 — Transcription factors predicted by WebGestalt to regulate differentially expressed genes in diclofenac and indomethacin treated cells. Transcription Factor Target Analysis of the differentially expressed gene sets was performed using WebGestalt (http://bioinfo.vanderbilt.edu/webgestalt/) to identify the transcription factors possibly involved in the regulation of the differentially expressed genes. Shown are the top transcription factors (highest significance) predicted along with the corresponding genes. Genes overexpressed are shown in bold and genes downregulated are shown in regular font. (TIF) [file pone.0061836.s002.tif]

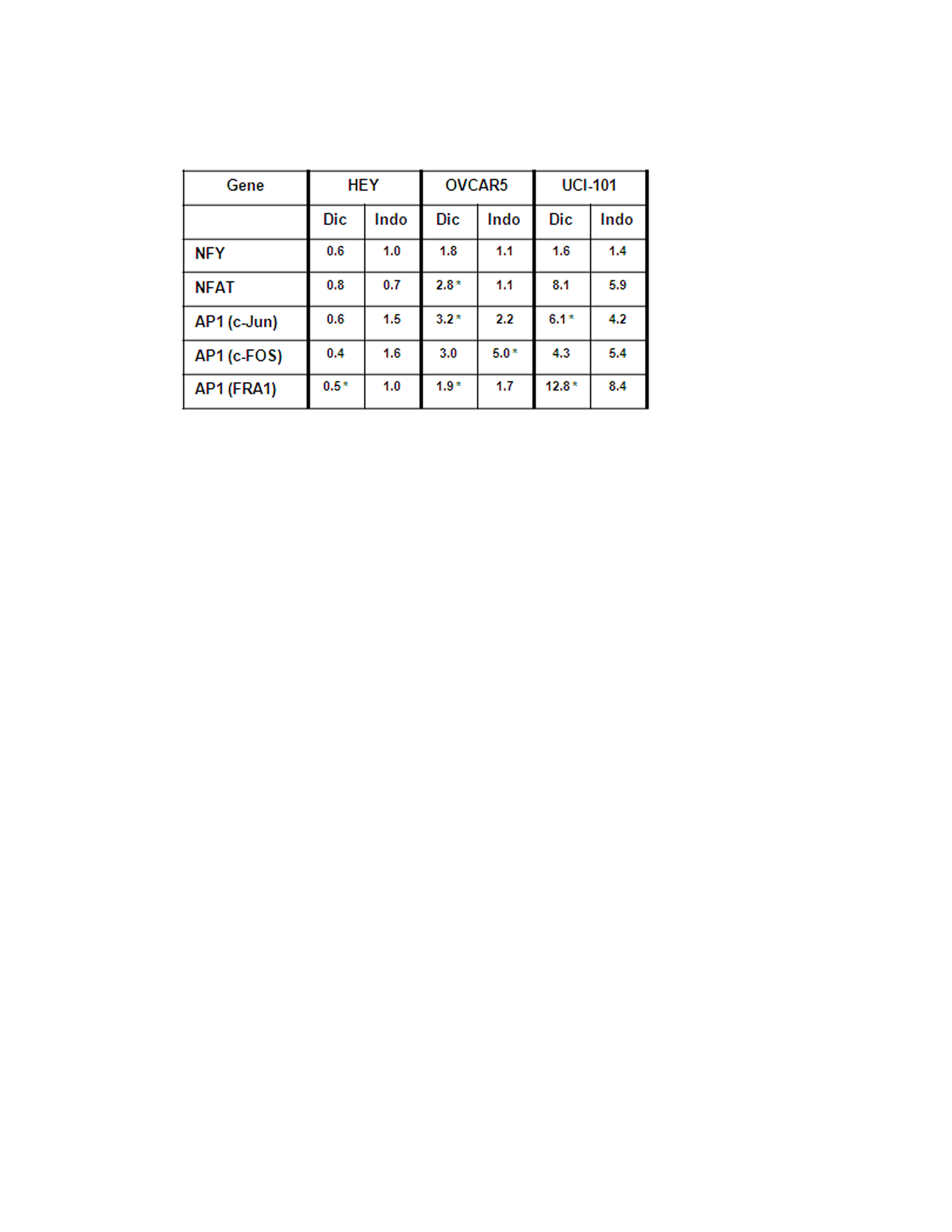

Supplement: Table S2 — Relative mRNA levels of transcription factors by Real-Time RT-PCR. Numbers reflect the fold change difference in the mRNA levels in the treated samples, as compared to the untreated samples, after normalization with GAPDH. Shown is the average of 3 independent experiments. One-way ANOVA and Dunnett's multiple comparison test was performed using Graph Pad Prism 3 software and statistical significance is represented as * p<0.05. (TIF) [file pone.0061836.s003.tif]
